# Supplementary material for: Practice and factors associated with active management of third stage of labor among obstetric care providers in Amhara region referral hospitals, North Ethiopia, 2018: A cross sectional study
Source: PLoS One. 2019 Oct 3;14(10):e0222843. doi: 10.1371/journal.pone.0222843 (PMC6776353; doi:10.1371/journal.pone.0222843)
Supplement: Attachment — Submitted filename: Comments on AMTSL in Ethiopia.docx [file pone.0222843.s002.docx]

Manuscript #: PONE-D-19-18594

**Title: Practice and factors associated with** **active management of third stage of labor among obstetric care providers in Amhara region referral hospitals, North Ethiopia, 2018: A cross sectional study.**

This cross sectional study which looked at the practice and factors associated with the active management of third stage of labor (AMSTL) among obstetric care providers in some referral hospitals in Africa is commendable and will add to the already existing body of knowledge on this topic. It is however limited by the fact that it only gives us a snapshot of these practices and factors.

The title of the study is appropriate, the sample size determination and methodology were systematic. The results showed that more than half of the respondents were actually practicing AMTSL and most of them were within the 20-30 years bracket. Being a male practitioner, having work experience ≥2 years, accessibility of oxytocin, exposure to third stage management and having good knowledge were some of factors associated with the practice.

The overall aim of this study was said to be “to fill the gaps and provide good intervention for care providers on practice and associated factor towards AMTSL at institution level”. This was however not written clearly under a separate heading. This study by the nature of it’s design may not be able to provide good intervention, the author may need to look at the aim again.

Under the Results, the labelling of the tables had the title of the whole study repeated under each table. My suggestion would be that Table 1 should be titled: Sociodemographic characteristics of the respondents, Table 2 should be: Provider, Institutional and Supplies related characteristics of respondents, and so on.

In Table 1, Under the Variables: in the subtitle of Qualifications; Senior Gynaecologist was used, I suggest that this be changed to Senior Obstetrician as this was an Obstetric study. Same goes for the subtitle of Profession where the word Gynaecologist was used instead of Obstetrician. Under the subtitle of Religion does Orthodox mean Catholic? Since the Protestants and Muslims both fall under orthodox religions. Medical Intern appeared under both Year of Graduation as well as under Profession. Please clarify.

Immediately after Table 1, under the sub heading “Provider, institutional and supplies/logistics related characteristics” in the first sentence, it was written that: “In this study 214(60.1%) of respondents were **taken AMTSL related training**”. This sentence is not clear. Do you mean the respondents had a previous training in AMTSL or are presently undergoing such training? Kindly clarify.

In Table 2: Under Variables: Does accessibility mean that these drugs were available for use at the time when it was needed? Accessibility is not synonymous to availability. Please explain.

In Table 3: “Types of Variables” was used as against ”Variables” used in Tables 1 & 2, please ensure consistency. Under the variable “Knowing the route of Oxytocin,” is it possible to know both the im and the iv routes? That option was not given.

Under Time of administration of uterotonic drugs; do you mean 1 minute/ 2-3 minutes of delivery of the baby? Clarify. Same in Table 4.

In Table 4 how was uterine relaxation ensured?

In the beginning of the second sentence in page 12 the following was written: “In multivariable logistic regression…..” Do you mean multivariate logistic regression? Kindly explain.

In Page 12 it is not clear where the discussion started from since there is a table immediately below the heading “Discussion”. The table should be under the results.

Table 5 is not clear. There should be a key to explain the single, double or triple asterix.

In page 13 in the second paragraph the author writes that: “Similarly this study showed that male obstetric care providers were 1.74 times more likely practiced AMTSL than females, which is different from other study. This might be due to assumption that females who have even the same opportunity on interaction with laboring mothers, males can have better interaction and they will act accordingly.” This sentence is not clear. Are women not more likely to be Nurses and Midwives hence have more opportunity to practice AMTSL? This particular sentence and few other sentences have some grammatic errors. I suggest the author works with a writing coach to improve this.

If the above issues raised are addressed by the author the editors can consider publishing the article.
